# The Interactive Effects of Race and Expert Testimony on Jurors’ Perceptions of Recanted Confessions

**DOI:** 10.3389/fpsyg.2021.699077

**Published:** 2021-09-03

**Authors:** Logan Ewanation, Evelyn M. Maeder

**Affiliations:** ^1^Department of Psychology, Carleton University, Ottawa, ON, Canada; ^2^Institute of Criminology and Criminal Justice, Carleton University, Ottawa, ON, Canada

**Keywords:** juror decision-making, recanted confessions, watchdog hypothesis, expert testimony, juries, confession evidence, defendant race

## Abstract

We examined the effect of defendant race and expert testimony on jurors’ perceptions of recanted confessions. Participants (591 jury-eligible community members) read a first-degree murder trial transcript in which defendant race (Black/White) and expert testimony (present/absent) were manipulated. They provided verdicts and answered questions regarding the confession and expert testimony. When examining the full sample, we observed no significant main effects or interactions of defendant race or expert testimony. When exclusively examining White participants, we observed a significant interaction between expert testimony and defendant race on verdicts. When the defendant was White, there was no significant effect of expert testimony, but when the defendant was Black, jurors were significantly more likely to acquit when given expert testimony. These findings support the watchdog hypothesis, such that White jurors are more receptive to legally relevant evidence when the defendant is Black.

## Introduction

Empirical research indicates that suspects falsely confess to crimes for a variety of reasons (Kassin and Kiechel, 1996; King and Snook, 2009). According to the Innocence Project (2021), false confessions^1^ were involved in approximately a quarter of the cases that have been exonerated through DNA evidence. However, confessions remain one of the most influential forms of evidence in the courtroom (Kassin and Neumann, 1997; Lieberman et al., 2008). Although expert witnesses are sometimes used to safeguard against issues with confession evidence, the effect of expert testimony on jurors’ perceptions of recanted confessions is unclear (Moffa and Platania, 2007; Blandon-Gitlin et al., 2011). Further, jurors’ perceptions of recanted confessions may depend upon the suspect’s race, as jurors perceive confessions as more voluntary when the defendant belongs to a racial minority (Pickel et al., 2013).

Most research focusing on the interaction between juror and defendant race has found that jurors are more lenient toward same-race defendants (see Devine and Caughlin, 2014). However, Sargent and Bradfield (2004) found that White mock jurors were more sensitive to legally relevant evidence in a trial transcript when the defendant was Black as compared to White. These authors argued that White jurors may attend to evidence more closely when the defendant is Black in an effort to serve as “watchdogs” against racism (termed the watchdog hypothesis). In a case in which a defendant has recanted their confession, the watchdog hypothesis would suggest that jurors may be more receptive to expert testimony (regarding factors that increase the likelihood of false confessions) when the defendant is Black, resulting in fewer guilty verdicts. The current study examines the interactive effects of defendant race and expert testimony on jurors’ perceptions of recanted confessions.

### Confession Evidence

Empirical research has demonstrated that individuals may falsely confess to crimes that they did not commit (e.g., Kassin and Kiechel, 1996; Redlich et al., 2010) for a variety of reasons, including coercive interrogation tactics (Kassin et al., 2003; King and Snook, 2009). However, in a criminal trial, a defendant’s confession is one of the most influential forms of evidence that the prosecution can present (Kassin and Neumann, 1997; Lieberman et al., 2008; Schweitzer and Nuñez, 2018). For instance, Lieberman et al. (2008) demonstrated that among several types of evidence, the only type that participants perceived as more persuasive than a suspect’s confession was DNA analysis. Even then, there are plenty of anecdotal instances in which law enforcement officials have ignored exculpatory DNA evidence in investigations when the suspect has confessed (e.g., the Central Park jogger case, Juan Rivera, the Norfolk Four; Duru, 2003; Leo and Davis, 2010). Furthermore, Appleby and Kassin (2016) conducted a series of jury studies involving conflicting DNA and confession evidence. Although participants were overall more likely to render a verdict in line with the DNA evidence, the authors also observed that perceptions of culpability and the proportion of guilty verdicts rose significantly when the prosecution presented a theory to explain the contradicting exculpatory DNA evidence (e.g., the DNA evidence only indicated that the defendant had not ejaculated).

Unfortunately, the phenomenon of false confessions is by no means a rare occurrence. Depending on particular definitions and methodology^2^, scholars estimate that false confessions contribute to 12–26% of wrongful convictions (Gudjonsson, 2003; Innocence Project, 2021; National Registry of Exonerations, 2020). Further research suggests that 73–81% of individuals who falsely confess are eventually convicted of the crime (Leo and Ofshe, 1998; Drizin and Leo, 2004). This type of evidence may be so problematic partly because jurors are unable to distinguish between true and false confessions (Kassin et al., 2005; Levine et al., 2010) and are unreceptive to the idea that an innocent person would ever falsely admit to a crime (Leo and Davis, 2010; Blandon-Gitlin et al., 2011). In an attempt to safeguard against the serious implications of a false confession, some states have allowed expert witnesses to testify about the science concerning this type of evidence (Kassin, 2008; Fulero, 2010).

### Expert Testimony

A small number of studies have investigated the effect that expert testimony has on jurors’ perceptions of recanted confessions. Jurors themselves report that such testimony would assist in their evaluation of this form of evidence (e.g., Chojnacki et al., 2008; Costanzo et al., 2010). However, findings concerning expert testimony’s actual influence on verdicts in confession trials are mixed (Blandon-Gitlin et al., 2011; Gomes et al., 2016; Henderson and Levett, 2016). For instance, a number of studies have observed no differences in verdicts between jurors who have and have not been presented with expert testimony in mock homicide trials (Moffa and Platania, 2007; Henderson and Levett, 2016, Study 2; Jones and Penrod, 2016). In comparison, Blandon-Gitlin et al. (2011) found that participants who read a trial transcript involving a recanted confession were less likely to render a guilty verdict after being exposed to expert testimony.

If working as intended, expert testimony should sensitize jurors to the quality of the confession (Cutler et al., 1989; Levett and Kovera, 2008), thus leading to fewer convictions when the confession is low quality (e.g., when several interrogation tactics known to elicit false confession have been used). However, rather than sensitizing jurors to variations in the quality of confession evidence, expert testimony may instead induce a general skepticism concerning confessions. For example, Woody and Forrest (2009) found that jurors presented with expert testimony concerning false confessions were significantly less likely to convict the defendant, regardless of whether a false evidence ploy was used in the interrogation. In a similar study involving mock jurors, Woestehoff and Meissner(2016, Study 3) manipulated the pressure and number of coercive interrogation tactics used in a defendant’s confession, as well as the presence of expert testimony. Overall, jurors were less likely to convict the defendant when exposed to expert testimony. This effect held regardless of how much pressure was involved in the defendant’s interrogation. Interestingly, Woestehoff and Meissner (2016) observed an effect of interrogation pressure independent of the expert witness; jurors given the high and medium-pressure conditions were significantly less likely to convict as compared to jurors who read about a low-pressure confession. These participants therefore appeared to be sensitive to variations in the confession evidence’s strength without the help of expert testimony.

Overall, there are conflicting findings regarding the effectiveness of expert testimony in cases involving a recanted confession. One factor that may explain these contradictory results is the race of the defendant. As described below, previous research has suggested that defendant race may influence jurors’ perceptions of the confession itself (Ratcliff et al., 2010; Pickel et al., 2013).

### Race in the Criminal Courtroom

An abundance of research examining the influence of defendant race has found that jurors often discriminate against defendants belonging to a racial minority (e.g., ForsterLee et al., 2006; Struckman-Johnson et al., 2008; Pickel et al., 2013). Specifically, jurors’ perceptions of confession evidence appear to depend upon the defendant’s race (Ratcliff et al., 2010; Pickel et al., 2013). Pickel et al. (2013) presented mock jurors with a confession video wherein the defendant’s race was ambiguous. Jurors who were told the defendant was Arab American rated the confession as more voluntary and were more likely to convict than those who believed the defendant was White. In similar research, Ratcliff et al. (2010) found that participants shown a video of a confession believed that the confession was more voluntary, and that the suspect was more likely to be guilty, if the suspect was Asian or Black, as opposed to White.

Several studies have further observed the existence of an overall similarity-leniency bias within the courtroom, such that jurors perceive defendants of the same race more favorably than other-race defendants (see Mitchell et al., 2005; Devine and Caughlin, 2014 for meta-analyses). This similarity-leniency bias may be explained by social identity theory (SIT; Tajfel and Turner, 1986), which argues that people have a motivation to favor and prefer individuals belonging to their groups (rather than those outside of their groups) as a method of promoting a positive self-concept. In a criminal trial, social identity theory would therefore predict that jurors are more likely to acquit a defendant of the same race (and more likely to convict a defendant of a different race).

However, the watchdog hypothesis (Sargent and Bradfield, 2004) suggests that White jurors are motivated to protect against discrimination and thus pay more attention to legally relevant information when the defendant is Black. Using simulated vignettes describing a robbery trial, Sargent and Bradfield (2004) manipulated alibi evidence strength (Study 1), cross-examination effectiveness (Study 2), and defendant race, in two samples of White mock jurors. They found that White jurors were more sensitive to manipulations of alibi strength and cross-examination effectiveness when the defendant was Black as opposed to White. The authors argued that White jurors may have attended to this information more closely in an effort to be “watchdogs” against racism. In trials involving recanted confessions, jurors may therefore be more receptive to expert testimony (concerning the phenomenon of false confessions) when the defendant is Black as compared to White.

### Current Study

Previous research has observed conflicting findings concerning the effect of expert testimony in trials involving recanted confessions. Furthermore, although research examining juror and defendant race has demonstrated a similarity-leniency bias (see Devine and Caughlin, 2014), Sargent and Bradfield (2004) suggest that White jurors may pay more attention to legally relevant evidence when the defendant is Black. The current study therefore aimed to examine the interactive effects of defendant race and expert testimony on jurors’ perceptions of recanted confessions. Drawing upon previous research, we developed two hypotheses.

**Hypothesis 1:** Based on previous literature suggesting that jurors perceive confessions to be less voluntary for White defendants (e.g., Ratcliff et al., 2010), and other research demonstrating an outgroup bias in verdict decisions (e.g., Devine and Caughlin, 2014), we predicted a main effect for defendant race such that [predominantly White (United States Census Bureau, 2019)] participants would be more likely to convict the Black defendant than his White counterpart.

**Hypothesis 2:** However, in accordance with the watchdog hypothesis (Sargent and Bradfield, 2004), we predicted an interaction between defendant race and expert testimony for White participants. Specifically, White participants would render fewer convictions in conditions with expert testimony as compared to conditions with no expert testimony for the Black defendant (with no such effect for the White defendant), as this testimony is legally relevant information to which they could attend to be “watchdogs” against racism.

## Materials and Methods

### Participants

Research has demonstrated that crowd sourced samples can be more heterogenous as compared to traditional undergraduate college samples (Paolacci et al., 2010; Paolacci and Chandler, 2014; Baker et al., 2016). We therefore recruited participants using Amazon’s Mechanical Turk (MTurk). We compensated participants with $3 for successfully completing the study. Although we had 1133 responses to our task, one participant did not give informed consent, 248 participants failed manipulation/attention checks^3^, 235 participants were ineligible for jury duty in the United States, and 58 participants quit the survey prior to completion. Our final sample therefore consisted of 591 jury-eligible community members (i.e., citizens of the United States who were at least 18 years old with no unpardoned felony conviction). Three-hundred and thirty-one (55.4%) of the participants were women, 263 (44.1%) were men, and three (0.5%) identified as another gender. Participants’ ages ranged from 19 to 69 years old (*M* = 36). Four-hundred and eighty-seven (81.6%) of the participants were White, 51 (8.5%) were Black, 27 (4.5%) were Hispanic, 21 (3.5%) were Asian, four (0.7%) were Native American, and seven (1.2%) identified as another race. Our participants’ racial demographics are similar to what other researchers have observed using MTurk (e.g., Burnham et al., 2018), and are comparable to the general United States population (United States Census Bureau, 2019), although our sample contained a slightly lower percentage of individuals identifying as Black (8.5 vs. 13.4%).

### Materials

#### Screening/Demographic Questionnaire

We used a demographic questionnaire in order to screen participants to ensure that they were jury-eligible. Participants were also asked to provide information regarding their race and gender.

#### Trial Transcript

We used a trial transcript adapted from previous research (Kassin and Sommers, 1997; Sommers and Kassin, 2001; Henkel, 2008). The transcript involved a defendant charged with murdering his wife and neighbor. The prosecution argued that the defendant had arrived home to find his wife and neighbor together, and believing they were having an affair, he killed them in an act of jealousy. However, the defendant claimed that his wife and neighbor were already dead when he came home. The defendant had initially confessed to the crime, but later recanted the confession. Apart from this confession, the remaining evidence was circumstantial (e.g., a witness saw someone fleeing the crime scene who matched the general physical description of the defendant). The defendant testified that during his interrogation he was handcuffed to a desk in a small room for more than 5 h and claimed the interrogating officer had physically threatened him with his service weapon. The defendant also stated that he was experiencing an immense amount of stress and in a state of shock during the interrogation because he had learned of his wife’s death only hours before. Finally, the defendant testified that the interrogating officer had repeatedly told him that his actions (killing his cheating spouse and her lover) were understandable, and that no one would blame him for what he did (i.e., minimization, Kassin and McNall, 1991). In each transcript, we manipulated the defendant’s race (Black, White) and presence of expert testimony (present, not present). Defendant race was manipulated by including a color photograph [matched in a pilot study (*N* = 30) on perceived age, likeability, and attractiveness] of the defendant, along with varying his name (Charles Smith for the White defendant and Jamaal Washington for the Black defendant) to strengthen our race manipulation. Previous research has used names to manipulate race (e.g., Bertrand and Mullainathan, 2004; Widner and Chicoine, 2011; Alhabash et al., 2014), as names can reinforce racial stereotypes and elicit biased judgments (Bodenhausen and Wyer, 1985; Watson et al., 2011; Garcia and Abascal, 2016). In half of the transcripts, an expert witness specializing in confession research testified for the Defense. The expert primarily testified about two situational factors – minimization techniques and extended periods of time – that increase the likelihood of a false confession, both of which he noted were present in the defendant’s confession and interrogation. The expert also discussed independent knowledge of the crime (underscoring the fact that the defendant’s confession did not include details that only the true perpetrator of the crime would know), as well as the prevalence of wrongful convictions that involve a false confession.

#### Jury Instructions

Before and after the transcript, we provided participants with juror instructions adapted from the California Criminal Jury Instructions (Judicial Council of California Civil Jury Instructions, 2020). The instructions discussed the criteria for first-degree murder, as well as the lesser-included second-degree murder and voluntary manslaughter charges, and also informed participants about the burden of proof and reasonable doubt.

#### Juror Questionnaire

In accordance with the legal instructions, we first asked participants to render a dichotomous verdict concerning the first-degree murder charge (guilty/not guilty). Participants who selected not guilty were then asked to render a dichotomous verdict concerning a second-degree murder charge (guilty/not guilty). Participants who still selected not guilty were then finally asked to render a dichotomous verdict concerning a voluntary manslaughter charge (guilty/not guilty). Logistically, we felt this method most appropriately reflected how jurors decide verdicts in California, as a juror who renders a guilty verdict for first-degree murder would not need to vote on the lesser-included offenses. We also asked participants to indicate on a scale from 1 (*not at all*) to 9 (*very much*) the degree to which they felt the defendant’s confession was voluntary (“How voluntary was the defendant’s confession?”).

The questionnaire also included a manipulation check, which asked participants to identify the race of the defendant from a list of options. In conditions with expert testimony, we asked participants to identify what the expert witness testified about (“What was a factor that the false confession expert, Dr. Turner, discussed?”) from a list of options to demonstrate that they had attended to this material. We embedded three other attention checks that required participants to select a specific response (e.g., “This is an attention check. Select Strongly Agree.”).

### Procedure

Participants were recruited from MTurk and completed the study online using Qualtrics survey software. Once participants had given informed consent, they were screened to ensure they met jury-eligibility requirements. We randomly assigned eligible participants to one of the four trial transcripts. Before and after reading the transcript, participants were provided with relevant legal instructions. After reading the transcript, participants responded to the juror questionnaire. Upon completion, participants were thanked, debriefed, and compensated. Participation in the study lasted approximately 30–45 min.

## Results

### Verdicts

Tables 1–3 display a breakdown of verdicts by condition for the first-degree murder, second-degree murder, and voluntary manslaughter charges, respectively. Table 4 summarizes the percentage of not guilty verdicts by verdict option for all participants as well as for White participants only. Prior to running the regression, we indicator coded our predictor variables (0 = no expert, 1 = expert present, and 0 = White defendant, 1 = Black defendant, respectively). We coded our ordinal outcome variable as 0 = not guilty, 1 = guilty of manslaughter, 2 = guilty of second-degree murder, and 3 = guilty of first-degree murder. To test our hypotheses, we conducted an ordinal regression with verdict being regressed on expert testimony, defendant race, and the interaction between expert testimony and defendant race. Results revealed no significant main effect of expert testimony, *b* = −0.04, OR = 0.96, 95% CI [0.64, 1.45], *W*^2^(1, *N* = 591) = 0.03, *p* = 0.861, or defendant race, *b* = −0.12, OR = 0.89, 95% CI [0.59, 1.34], *W*^2^(1, *N* = 591) = 0.33 *p* = 0.568. Additionally, the expert testimony by defendant race interaction was non-significant, *b* = 0.56, OR = 1.75, 95% CI [0.97, 3.15], *W* (1, *N* = 591) = 3.48, *p* = 0.062.

**TABLE 1:** First-degree murder verdicts by defendant race and expert testimony.

Defendant race	Expert testimony	Verdict	Frequency	Percent
White	No Expert Testimony	Not guilty	113	72.9%
		Guilty	42	27.1%
		Total	155	100.0%
	Expert Testimony	Not guilty	105	72.9%
		Guilty	39	27.1%
		Total	144	100.0%
Black	No Expert Testimony	Not guilty	96	68.1%
		Guilty	45	31.9%
		Total	141	100.0%
	Expert Testimony	Not guilty	120	79.5%
		Guilty	31	20.5%
		Total	151	100.0%

**TABLE 2:** Second-degree murder verdicts by defendant race and expert testimony.

Defendant race	Expert testimony	Verdict	Frequency	Percent
White	No Expert Testimony	Not guilty	75	66.4%
		Guilty	38	33.6%
		Total	113	100.0%
	Expert Testimony	Not guilty	77	73.3%
		Guilty	28	26.7%
		Total	105	100.0%
Black	No Expert Testimony	Not guilty	68	71.6%
		Guilty	27	28.4%
		Total	95	100.0%
	Expert Testimony	Not guilty	89	74.2%
		Guilty	31	25.8%
		Total	120	100.0%

**TABLE 3:** Voluntary manslaughter verdicts by defendant race and expert testimony.

Defendant race	Expert testimony	Verdict	Frequency	Percent
White	No Expert Testimony	Not guilty	60	80.0%
		Guilty	15	20.0%
		Total	75	100.0%
	Expert Testimony	Not guilty	51	66.2%
		Guilty	26	33.8%
		Total	77	100.0%
Black	No Expert Testimony	Not guilty	52	76.5%
		Guilty	16	23.5%
		Total	68	100.0%
	Expert Testimony	Not guilty	70	78.7%
		Guilty	19	21.3%
		Total	89	100.0%

**TABLE 4:** Percentage of not guilty verdicts by charge.

Charge	% of Not guilty verdicts	
	Full sample	White participants
First-degree murder	72.6%	74.5%
Second-degree murder	52.0%	53.3%
Voluntary manslaughter	39.3%	40.5%

Because the watchdog hypothesis specifically involves White individuals (Sargent and Bradfield, 2004), we re-ran our initial regression using only White participants (*N* = 482). As before, we conducted an ordinal regression on verdict using expert testimony, defendant race, and the interaction as the predictor variables. Again, we observed no significant main effects of expert testimony, *b* = −0.26, OR = 0.77, 95% CI [0.49, 1.21], *W*^2^(1, *N* = 482) = 1.29, *p* = 0.257, or defendant race, *b* = −0.35, OR = 0.70, 95% CI [0.44, 1.11], *W*^2^(1, *N* = 482) = 2.26, *p* = 0.133. However, there was a significant interaction between expert testimony and defendant race, *b* = 0.93, OR = 2.56, 95% CI [1.33, 4.92], *W*^2^(1, *N* = 482) = 7.9, *p* = 0.005. We probed this interaction for White participants by running two separate ordinal regressions with expert testimony as the predictor, splitting the data file based on defendant race. Analyses indicated that for White jurors, there was no effect of expert testimony when the defendant was White, *b* = −0.26, OR = 0.77, 95% CI [0.49, 1.21], *W*^2^(1, *N* = 245) = 1.28, *p* = 0.258. In comparison, we observed a significant effect of expert testimony when the defendant was Black, *b* = 0.68, OR = 1.98, 95% CI [1.23, 3.17], *W*^2^(1, *N* = 237) = 8.04, *p* = 0.005. The odds of White jurors rendering a not guilty verdict (versus other verdict options) for the Black defendant were approximately twice as high when given expert testimony as compared to when no such testimony was presented.

Using the hmisc package (Harrell, 2021) in R (R Core Team, 2021), we conducted a *post hoc* sensitivity analysis to provide an estimate of the smallest effect size that we would have sufficient power (i.e., 80%) to detect. Analysis indicated that for an overall N of 590, our design had a power of.80 to detect an odds ratio of 1.52, which is equivalent to a “small” effect size under Cohen’s conventions (see Chen et al., 2010). Therefore, we appeared to be sufficiently powered to conduct our ordinal analyses.

### Voluntariness of Confession

We conducted an exploratory analysis on participants’ perceptions of how voluntary the defendant’s confession was. We were interested in examining effects on voluntariness in particular because we felt this was a purer measure of jurors’ perceptions of the confession itself. In comparison, participants’ final verdicts could be influenced by a number of factors unrelated to the confession evidence (e.g., the circumstantial evidence presented at trial).

Overall, participants scored near the midpoint on their perceived voluntariness of the defendant’s confession (*M* = 4.71, *SD* = 2.57). We ran a 2 × 2 ANOVA to test the degree to which defendant race and expert testimony influenced this rating. Results revealed a significant main effect for expert testimony [*F*(1,586) = 4.76, *p* = 0.03, η*^2^_*p*_* = 0.008, *w*^2^*_*p*_* = 0.006]; participants who received expert testimony perceived the defendant’s confession to be less voluntary (*M* = 4.48, *SD* = 2.50) than those who did not (*M* = 4.94, *SD* = 2.62). The main effect for defendant race was not significant [*F*(1,586) = 0.14, *p* = 0.71, *partial*η*^2^* = 0.001 *w*^2^*_*p*_* ≤ 0.001], nor was the interaction [*F*(1,586) = 2.17, *p* = 0.14, η*^2^_*p*_* = 0.004, *w*^2^*_*p*_* = 0.002].

**FIGURE 1:**
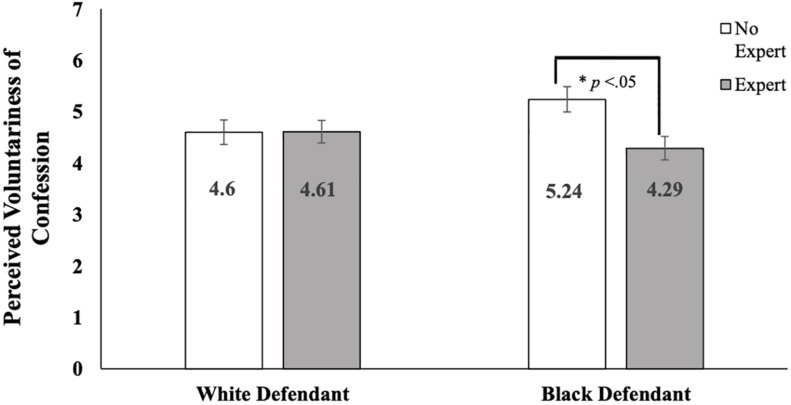
Ratings of voluntariness of confession by defendant race and expert testimony for White participants.

As above, we re-ran this analysis with only White participants. This test again revealed a small, significant main effect for expert testimony [*F*(1,477) = 4.00, *p* = 0.046, η*^2^_*p*_* = 0.008, *w*^2^_*p*_ = 0.006], qualified by a significant interaction between defendant race and expert testimony [*F*(1,477) = 4.20, *p* = 0.041, η*^2^_*p*_* = 0.009, *w*^2^*_*p*_* = 0.007]; the main effect for defendant race was not significant [*F*(1,477) = 0.46, *p* = 0.50, *partial*η*^2^* = 0.001, *w*^2^*_*p*_* ≤ 0.001]. To probe the interaction, we first compared the effects of expert testimony on voluntariness ratings by defendant race. Simple effects tests demonstrated that for those who read about a White defendant, voluntariness ratings did not differ significantly in the presence (*M* = 4.61, *SD* = 2.45) or absence (*M* = 4.60, *SD* = 2.66) of expert testimony, *t*(242) = −0.04, *p* = 0.98, *d* = 0.01, 95% CI [−0.25,0.24]. However, participants who read about a Black defendant were significantly less likely to perceive his confession as voluntary when they received expert testimony (*M* = 4.29, *SD* = 2.52) as compared to when they did not (*M* = 5.23, *SD* = 2.63), *t*(235) = 2.83, *p* = 0.005, *d* = 0.37, 95% CI [0.11,0.63]. See Figure 1 for a visual display of this relationship. When probing the interaction the other way, we did not find significant effects for defendant race in either the expert testimony present {*t*(240) = 1.01, *p* = 0.315, *d* = 0.13, 95% CI [−0.12,0.38]} or absent {*t*(237) = −1.86, *p* = 0.064, *d* = 0.24, 95% CI [−0.50,0.01]} conditions.

## Discussion

The aim of this study was to explore the combined effects of defendant race and expert testimony on jurors’ decision-making in trials involving a recanted confession. In line with the watchdog hypothesis (Sargent and Bradfield, 2004), when the defendant was Black, White jurors were significantly more likely to render a not guilty verdict when given expert testimony. In comparison, White jurors’ verdicts were not significantly influenced by expert testimony in conditions involving White defendants. This same pattern was found for perceptions of the confession’s voluntariness, although findings relating to the voluntariness measure require further confirmatory testing from future research.

Our results may demonstrate evidence of a sensitivity effect in situations involving a Black defendant and a confession. Given that jurors in the Black defendant condition convicted less often and perceived the confession as less voluntary, these jurors likely attended more to the expert testimony than did those in the White defendant condition. Doing so may have allowed the testimony to sensitize jurors to the issues related to the disputed confession (e.g., long period of time, minimization and maximization techniques employed, lack of independent knowledge of the crime, etc.). However, because we did not manipulate the strength of the confession, it is unclear whether the expert testimony truly sensitized jurors in these conditions. Instead, they may have simply become skeptical of all confession evidence. Given that earlier findings regarding expert testimony and confession evidence suggest that the mere presence of expert testimony (regardless of the presence of coercive interrogation tactics) could reduce reliance on confessions (Woody and Forrest, 2009), this was an important first step in establishing the presence of a watchdog effect. Future research should extend the current study’s design with the inclusion of a confession quality (i.e., lower vs. higher number of coercive tactics present in the interrogation) manipulation. Doing so would allow for a better understanding of whether sensitivity or skepticism is occurring in these situations.

Because our sample was predominantly White, we predicted an overall similarity-leniency bias such that Black defendants would be more likely to be convicted than defendants who were White (Devine and Caughlin, 2014). Contrary to predictions, there was no significant main effect of defendant race on jurors’ verdicts. Although this finding conflicts with research supporting the similarity-leniency hypothesis (e.g., Ugwuegbu, 1978; Sommers and Ellsworth, 2000; Devine and Caughlin, 2014), other research has also demonstrated null effects relating to defendant race (e.g., Braden-Maguire et al., 2005; Maeder et al., 2012; Yamamoto and Maeder, 2017).

There are a number of potential explanations for why we observed no significant overall effect of defendant race. According to the aversive racism framework (Schweitzer and Nuñez, 2018), the influence of racial bias is greatest in ambiguous situations (Dovidio and Gaertner, 1996,2000, 2004). In the current study, the legal instructions likely removed some of the ambiguity concerning participants’ verdict decision, lessening the effect defendant race may have had (Pfeifer and Ogloff, 1991). Furthermore, a recent meta-analysis observed out-group bias in studies involving property crimes or adult sexual assaults, but much smaller (or non-existent) effects in studies using violent cases (Devine and Caughlin, 2014). Because our trial transcript involved a murder, future research should consider replicating the current study using other crimes, such as burglary.

Finally, our data were collected between June and December 2018. During this time, the police’s unjust treatment of Black individuals became a salient topic in the media (e.g., Carney, 2016; Lopez, 2018; Scott, 2018). Our participants may therefore have been cognizant of the potential for such discrimination, particularly because the defendant claimed that he was threatened and coerced by police during his interrogation. Previous research has found that White jurors’ bias against BIPOC defendants is minimized when racial issues are made salient during the trial (e.g., Cohn et al., 2009; Bucolo and Cohn, 2010). It is a strong possibility that cases involving alleged police misconduct are inherently race salient, leading jurors to correct for racial bias and resulting in a null effect of defendant race (Sommers and Ellsworth, 2000, 2001). It is important to note that following the death of George Floyd in May 2020, the topic of racial discrimination in the United States’ justice system received unprecedented attention and media coverage. We encourage researchers to replicate and extend these findings to see what effect these recent events may have had in this context.

Similar to our results concerning defendant race, we observed no significant main effect of expert testimony on jurors’ verdicts. This complements the work of Jones and Penrod (2016), as well as Moffa and Platania (2007), but contradicts a number of other studies that did observe an effect of expert testimony in trials involving recanted confessions (Woody and Forrest, 2009; Blandon-Gitlin et al., 2011; Woestehoff and Meissner, 2016). Related research concerning jurors’ perceptions of secondary confessions has also observed no significant effect of expert testimony on verdicts (Neuschatz et al., 2012; Maeder and Pica, 2014).

In comparison to our results concerning expert testimony and verdict, there was a significant main effect of expert testimony on perceived voluntariness of the confession. One explanation for this pattern may be that although expert testimony lowered jurors’ perceived voluntariness of the confession, they still viewed the confession itself as indicative of guilt. Researchers have used the fundamental attribution error to explain jurors’ reluctance to discount disputed confession (e.g., Kassin and Sukel, 1997; Kassin and Gudjonsson, 2005). In our study, jurors may indeed have perceived the confession as less voluntary following expert testimony, but they still may have believed that overall, the defendant confessed because he was guilty (rather than because of the situational factors present). In similar research, Kassin and Wrightsman (1981) found instructions on the unreliability of coerced confessions significantly decreased participants’ perceived voluntariness of the confession, but did not influence verdicts.

In their work, Sargent and Bradfield (2004) manipulated the strength of the defendant’s alibi as well as the strength of the prosecutor’s cross-examination^4^; future research should continue to examine the watchdog hypothesis by manipulating other types of evidence and/or expert testimony (such as expert testimony concerning police use of force or eyewitness identifications). Because the watchdog effect has now been demonstrated using both direct evidence (i.e., defendant’s alibi) as well as trial-level phenomena (i.e., cross-examination and expert testimony), we tentatively predict that our observed effects would likely generalize to these other forms of evidence. Further, our results underscore the notion that there is a complex effect of race in the courtroom that goes beyond a simple similarity-leniency effect; we found White jurors to be more lenient to the racial outgroup when given expert testimony. As discussed above, it may be the case that, due to the increased public attention regarding racial discrimination in the legal system, the similarity-leniency effect is minimized (or outright reduced) in trials involving potential police misconduct. More work examining this issue, particularly sampling from BIPOC jurors, is needed to better understand these complexities. Based on these preliminary results, White jurors appear to either interpret or apply evidence differently depending upon the defendant’s race, ultimately leading to different verdict decisions. Specifically, our findings suggest that attorneys should particularly consider the use of expert testimony in trials involving a BIPOC defendant and a recanted confession.

Finally, although we found evidence to suggest a watchdog effect, there are other possible explanations for our findings. In our study, White participants may have been more likely to use expert testimony in their verdict decisions when the defendant is Black as opposed to White not because they are paying more attention to legally relevant factors (as per the watchdog hypothesis), but because they are looking for a reason to acquit the Black defendant. This may be in an attempt to establish non-racist credentials (e.g., Effron and Conway, 2015) – when evaluating a Black defendant, White participants may feel as though their moral standing is uncertain, and so make greater use of the expert testimony and subsequently acquit in order to demonstrate their egalitarianism. Future research could implement a detailed measure of comprehension of the expert’s testimony. This would reveal whether participants comprehend the information better when the defendant is Black, or whether they simply use the expert testimony as a reason to acquit the Black defendant.

### Limitations

Our study’s methodology had a number of limitations. To begin, we used a written trial transcript, which limited ecological validity (Wenger and Bornstein, 2006). However, existing literature suggests that presentation mode does not significantly affect mock jurors’ verdict decisions (Bornstein, 1999; Pezdek et al., 2010). Furthermore, our participants were likely aware that their responses had no true consequences, which may have influenced our findings (Bornstein and McCabe, 2005; Bornstein et al., 2017). Studying real jury trials would help to overcome this problem of consequentiality and may have led to different results. However, such a methodology would also introduce a host of additional issues regarding feasibility and internal validity. Like most jury research, we also only used a single trial transcript that had specific evidence and charges. Replications using additional cases would increase the generalizability of our results.

An additional ecological limitation of the current study is the lack of a deliberation component. Although research has demonstrated that the jury’s final verdict is often predicted from individual verdicts (Kalven and Zeisel, 1966; Devine et al., 2007), other literature suggests that deliberation can influence jurors’ bias (London and Nunez, 2000) and also affect jurors’ cognitive processes when trying to reach a decision (Salerno and Diamond, 2010; Salerno et al., 2017). Sommers (2006) has further demonstrated that the racial composition of a jury can influence how jurors talk about race, which may be relevant to our results as our study involved a Black defendant in half of the conditions. Therefore, future research examining the watchdog hypothesis should likely involve a deliberation component.

Because the study was conducted online on MTurk, there was a lack of general control over the environment in which participants responded, which may have produced environmental confounds. However, we implemented manipulation and attention checks to ascertain data quality (e.g., Peer et al., 2014). Using MTurk also allowed for recruitment from a nationwide community sample (rather than relying on an undergraduate sample from a single university), which likely increased the generalizability of our results (Baker et al., 2016). Regardless, we still had a fairly racially homogenous sample, as response rates from BIPOC participants were low. We were therefore unable to do any proper comparisons based on juror race. Although the watchdog hypothesis focuses specifically on White jurors, future research needs to be conducted that explicitly examines BIPOC jurors’ perceptions in the courtroom.

## Conclusion

Our study examined the role of defendant race and the influence of expert testimony in the context of trials involving recanted confessions. To the best of our knowledge, this is the first study to examine the interactive effects of these variables. For White jurors, we observed an interaction between defendant race and the presence of expert testimony. There was no significant effect of expert testimony on verdict when the defendant was White, but White jurors were significantly less likely to find the Black defendant guilty (and perceive his confession as voluntary) when presented with expert testimony concerning false confessions. These findings support the existence of the watchdog hypothesis (Sargent and Bradfield, 2004), such that White jurors are more receptive to legally relevant evidence when the defendant is Black. To gain a stronger understanding of when this effect is elicited, future research should replicate the current study using other types of evidence and expert testimony.

## Data Availability Statement

The raw data supporting the conclusions of this article will be made available by the authors, without undue reservation.

## Ethics Statement

The studies involving human participants were reviewed and approved by the Carleton University Research Ethics Board-B (CUREB-B) Carleton University. The patients/participants provided their written informed consent to participate in this study.

## Author Contributions

LE and EM contributed to the conception and design of the study and conducted the data analysis. LE organized the data collection and wrote the first draft of the manuscript. EM wrote the sections of the manuscript. Both authors contributed to manuscript revision, read, and approved the submitted version.

## Conflict of Interest

The authors declare that the research was conducted in the absence of any commercial or financial relationships that could be construed as a potential conflict of interest.

## Publisher’s Note

All claims expressed in this article are solely those of the authors and do not necessarily represent those of their affiliated organizations, or those of the publisher, the editors and the reviewers. Any product that may be evaluated in this article, or claim that may be made by its manufacturer, is not guaranteed or endorsed by the publisher.

## Footnotes

1 The Innocence Project includes false admissions and other forms of self-incrimination in their definition of false confessions.

2 The National Registry of Exonerations defines a false confession as “a statement made to law enforcement at any point during the proceedings which was interpreted or presented by law enforcement as an admission of participation in or presence at the crime, even if the statement was not presented at trial. A statement is not a confession if it was made to someone other than law enforcement. A statement that is not at odds with the defense is not a confession. A guilty plea is not a confession.” In comparison, the Innocence Project’s definition includes false admissions and other forms of self-incrimination in their definition of false confessions.

3 Sixty-two participants failed the manipulation check concerning defendant race, while 184 participants failed the manipulation check concerning the content of the expert’s testimony. For attention checks, participants were instructed to select a particular response. Twenty-six participants failed the first attention check, 29 participants failed the second attention check, and 33 failed the third attention check.

4 In the “weak” cross-examination condition, participants read a case summary where the court reporters indicated the prosecution had presented ineffective cross-examinations of the defense witnesses. In the “strong” condition, the case summary described the prosecution as presenting an effective cross-examination that diminished the defense witnesses’ credibility.
